# The Function of the Cecum and Appendix

**Published:** 1905-01

**Authors:** 


### THE FUNCTION OF THE CECUM AND APPENDIX.

The large amount of study which has been applied to the vermiform appendix in the last ten years has had to do, in the main, with the surgical aspects of this organ. It is true that the widespread recognition of the importance of this structure in human pathology has led to a restudy of its anatomy and pathology, but the physiology has been greatly neglected. In his recent Huxley Lecture Macewen* calls attention to these facts: He states that, as a rule, the appendix is described as a rudimentary organ, and that some writers regard even the cecum as a useless inheritance derived from our prehistoric ancestors. He comments on the modern tendency to regard organs whose functions we do not fully understand as useless organs, and the resulting idea that it is of benefit to remove such organs, even when not diseased, when they are exposed by operations done for other purposes. He suggest the improbability that Nature has constructed our bodies so faultily that it is necessary for us to patch and trim them, as is often suggested and occasionally done. He then attempts to show that the cecum and appendix have useful functions, and are not the useless structures that the appendectomaniacs would have us believe.

He considers the subject first from the comparative view-point, and shows that the relative importance of the stomach and the cecum in the digestive process is determined by the kind of food on which the animal lives. In carnivorous birds and animals most of the work of digestion is done by the stomach, and the cecum is small or rudimentary; in herbivora, on the other hand, the cecum is enormously developed, and in certain species, the solipeds, the cecum is the chief digestive organ. This suggests, Macewen states, that man, who is both carnivorous and herbivorous, should possess not only a stomach but also a cecum, and this is indeed the case. The relatively small size of the cecum in man can be explained by the fact that the vegetables which he consumes are soft and relatively easily digested.

Observations on human beings which bear on the function of the cecum and appendix have been obtained as a result of injury or of operation, and are necessarily somewhat fragmentary. The effects of fecal fistulæ occurring at various levels in the intestines have shown in a general way that the nearer a fistula is to the

* *British Medical Journal*, October 8, 1904.

stomach the more pronounced are the symptoms of malnutrition. Where a fecal fistula is situated just above or in the cecum, provided in the latter case that the opening be large, a certain amount of interference with nutrition results. In patients in whom the cecum has been removed the digestive processes are practically never normal, and all of these cases have to live on a special diet. In the few cases in which an opening in the cecum has existed which was large enough to allow of easy inspection, certain interesting occurrences have been observed. It has been noted that certain definite movements occur in the cecum as a result of stimulation by food, and it has also been noted that one or two hours after the introduction of food into the stomach there is a considerable secretion of mucus by both the cecum and the appendix. Then, too, it has been noted in such cases that the ileocecal valve allows only the intermittent passage of the chyme, a circumstance which would favor its admixture with the cecal and appendiceal secretions. Anatomically, the situation of the valve opening just above the appendiceal opening, the dependent situation of the cecum, and the presence of large numbers of Lieberkühn in the cecum and appendix, all speak for something more than a mere mechanical action.

The bearing of these facts on appendicitis Macewen merely touches on, and in this part of his paper he really brings out nothing which we had not already learned empirically. The relation of appendicitis to digestive disturbances and the preventive measures which logically suggest themselves were already sufficiently obvious. The first part of the paper, however, is very suggestive, and should be read by the surgeons and gynecologists, and especially by those who advocate removal of the normal appendix whenever an opportunity offers.—*Journal American Medical Association.*

